# The iliac stemmed cup in reconstruction of the acetabular defects secondary to tumor resection: a systematic review of literature

**DOI:** 10.1007/s00402-022-04639-3

**Published:** 2022-10-01

**Authors:** Carmine Zoccali, Giuseppe Giannicola, Giovanni Zoccali, Elisa Checcucci, Alessandra Scotto di Uccio, Dario Attala, Ciro Villani

**Affiliations:** 1 Department of Anatomical, Histological, Forensic Medicine and Orthopaedic Science, University of Rome, Piazzale Aldo Moro 5, 00185 Rome, Italy; 2 Oncological Orthopaedics Department, IRCCS – Regina Elena National Cancer Institute, Via Elio Chianesi 53, Rome, Italy; 3 Department of Plastic and Reconstructive Surgery, IRCCS – Regina Elena National Cancer Institute, Via Elio Chianesi 53, Rome, Italy; 4 Hepato-Biliary and Organ Transplant Unit, School of General Surgery, Sapienza University, Viale del Policlinico 155, 00161 Rome, Italy; 5 Orthopaedic and Traumatology Unit, Department of General Surgery, Plastic Surgery, Orthopedics, Policlinico Umberto I Hospital-Sapienza, University of Rome, Piazzale A. Moro 3, 00185 Rome, Italy

**Keywords:** Stemmed acetabular cup, Hip prosthesis, Hip revision, Pelvis tumor

## Abstract

**Introduction:**

Stemmed acetabular cups are suitable for reconstruction in case of important bone loss. Nevertheless, their use is not so common, because generally judged very invasive and technically difficult to implant. The aim of the present review is to verify the results of their use and to evaluate indications and complications.

**Materials and methods:**

Literature research was performed in the main healthcare databases; indications, surgical technique, related complications, functional results and implant survival were valued and analyzed for every selected paper.

**Results:**

13 studies were selected, for a population of 424 patients and 428 hips. The main indication was reconstruction after tumor removal; the primary non-oncologic indication was revision for aseptic loosening. The most frequent complications were aseptic loosening and implant failure (16.2%), followed by deep infection (11.3%) and dislocation (9.8%). The average MSTS score was 65.9%; while data regarding functional results for degenerative cases are quite fragmented. The 5-years implant survival was 73.6%.

**Conclusions:**

Data regarding SAC prostheses are quite rare in the literature; no prospective studies with comparisons with other reconstruction techniques are available so their use is mainly based on the experience of single centers. While data for tumors are more consistent and supported by studies, information on revisions of hip prosthesis implanted for degenerative problems is quite scarce. Preliminary results on the SAC prosthesis as a valid alternative both for tumoral and degenerative revision cases are encouraging. Prospective randomized studies are advocated to value results compared to alternative techniques.

## Introduction

In hip prostheses, acetabular fixation is usually obtained through press-fit with the surrounding bone; this is easily achieved in young patients with good bone quality; in the elderly, this is not always possible and cementing the acetabular component can sometime be necessary.

In case of important osteolysis due to degenerative problems, bone resorption, multiple revisions or after tumor resection, a press-fit does not guarantee stability so the use of more invasive acetabular system as modular acetabulum, custom-made components or stemmed acetabular cups (SAC) could be necessary [1–3]. SAC present a stem which is inserted into the ileum, giving primary stability.

SAC use is not so common, maybe because it is considered very invasive and technically difficult; however, the supposed difficulty is mostly linked to the low number of cases performed while the invasiveness is not higher than a standard reconstruction acetabular cap, especially considering that it can be used with bone grafting to restore the bone stock.

Based on these considerations, the aim of the present systematic review is to verify results of its use in reconstruction of acetabular defects, and to evaluate indications and complications.

## Materials and methods

This review was performed in accordance with the Preferred Reporting Items for Systematic Reviews and Meta-Analyses (PRISMA) guidelines and was completed in May 2022 [4].

### Strategy search

An electronic search was performed in healthcare databases including MEDLINE, EMBASE, SCOPUS, and the Cochrane library using the keywords “Cone Acetabular Prosthesis”, “Iliac Stem”, “Ice-Cream Cone Prosthesis”, “Stemmed Acetabular Cup” and “McMin Cup”, looking for prospective and retrospective papers published in English without date restriction, analyzing results of the iliac stemmed cup prosthesis in reconstruction of acetabular defects after tumor treatment (both intralesional and wide treatment).

Exclusion criteria were population inferior to ten cases or confused records; papers not reporting exhaustive data were excluded; cases with follow-up inferior to 1 year were also excluded from the series; when the identification was not possible, the entire study was not included.

In case of papers published by the same research group on a similar population, the texts were carefully analyzed and only the most recent paper was included in the review to decrease the risk of considering the same patients twice or more.

### Study selection

After reviewing the resultant abstracts, two independent reviewers selected the papers; further selection was performed based on the entire paper and on the pre-established criteria. References were also screened to find other possible inclusions.

In cases of disagreement between the reviewers regarding paper inclusion, a consensus was reached.

### Data extraction and outcome measures

For each selected study, we evaluated epidemiological characteristics, diagnosis, status of the disease (primary or local recurrence), surgery, related complications, follow-up and clinical status.

Scales and scores applied in the selected studies were valued; for oncological studies, the Muscular-Skeletal Tumor Society (MSTS) score was considered as reference scale to value functional results; a score > 23 was considered “excellent result”; between 15 and 22 “good result”, between 8 and 14 “fair result”, and inferior to 8 “poor result” [5].

### Statistical method

A descriptive analysis including clinical and demographic characteristics of the patients was performed through median and range for continuous variables and absolute value and relative frequencies for categorical variables. The association between variables was tested by the Pearson Chi-Square.

No funding was received for the publication of the present paper; the authors declare no conflict of interest.

## Results

**Fig. 1:**
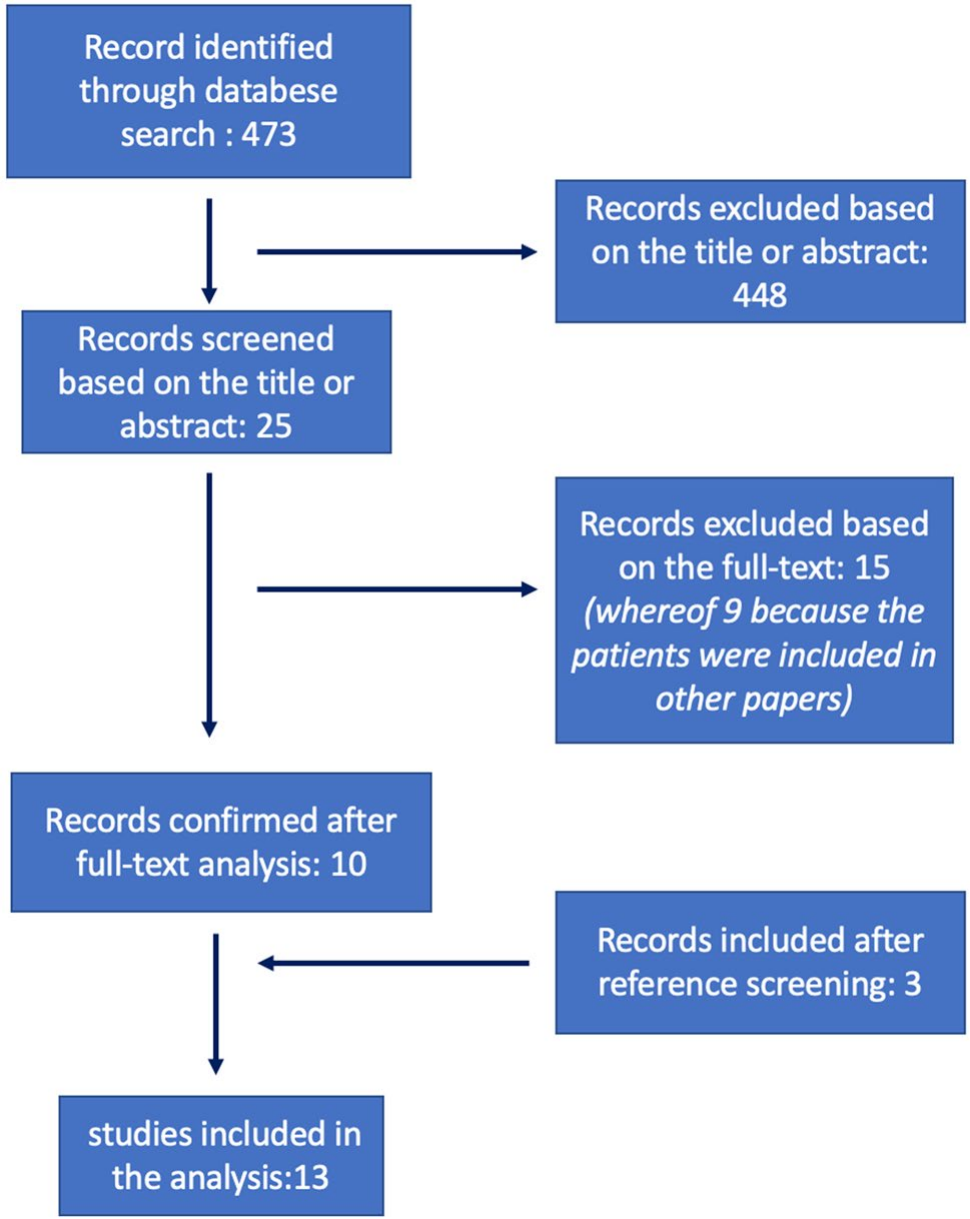
Flowchart showing the selection process

The first electronic search identified 473 papers. After excluding recurrent titles, the first screening was performed based on titles and, in case of doubt, on abstracts and 25 papers were selected. These papers were carefully analyzed based on the full texts and 15 were excluded, whereof 9, because their patients were already included in other more recent publications. Subsequently, three papers were included, while screening the bibliographies of the remaining ten papers. At the end of the process, 13 papers were included in the review (Fig. 1).

### General population

13 studies were selected, for a population of 424 patients (176 males and 248 females) and 428 hips, with an average age of 61.4 years (Table 1).

**Table 1:** Epidemiological and clinical characteristics of selected papers’ populations

Papers	Pts	M/F	age	Diagnosis	Paprosky classification	Enneking and Dunham	Histologies	SAC
Mallet et al. [6]	56	12/44	71.7 (± 15.1)	ST: 19; PF: 12; AF: 3; AL: 14; SL: 8	1: 1; 2A: 0; 2B: 3; 2C: 9; 3A: 24; 3B: 19	nr	nr	Integra Lepine (Genay France)
	31	9/22	72.0 (± 13.8)	ST: 12; PF: 7; AF: 2; AL: 4; SL: 6	nr	nr	nr	Integra Lepine (Genay France)
	25	3/22	71.4 (± 16.3)	ST: 7;PF: 5; AF: 1;AL: 10; SL: 2	nr	nr	nr	Integra Lepine (Genay France)
Fujiwara et al. [7]	54	27/27	58 (13–82)	PT; ST	na	II: 31; II/III: 10; Ip-II: 5; Ip- II–III: 5; I–II–III: 1; I–II–IV: 2	CS: 18; OS: 4; ES: 2; EH: 1; ST: 25; HT: 4	Stanmore
Issa et al. [8]	14 (16 hips)	9/5	72.8 (58–95)	AL	IIIa:7, IIIb:9	na	na	Integra Lepine (Genay France)
McMahon et al. [9]	21 (22 hips)	11/10	79 (67–87)	AF	nr	na	na	Stryker
Erol et al. [10]	21	11/10	47 (± 16)	PT; ST	nr	II: 14; I + II + III: 2; II + III: 5	CS: 10; EW:6; ST: 5	Lumic Implant Cast
Issa et al. [11]	24	10/14	46 (18–75)	PT; ST; R of T	na	PT II: 4; II–III: 12 R II: 3 II + III: 4	CS: 10 (1 dedif, 5 classic G2, 3 myxoid, 1 periosteal G2) MVS:1 OS: 4 ST: 2 AL in (MS: 1; EW: 1; OS: 3; CS: 2)	Integra Lepine (Genay France)
Bus et al. [12]	47	26/21	50 (12–78)	PT; ST	na	type II: 21—Type II/III: 26	CS G2 or G3: 13; ST: 5; OS: 5; ES: 4; CS G1: 4; MM: 3; PUS: 1; SNAS: 1; PMT: 1 R of T: 9	Lumic Implant Cast
Stihsen et al. [13]	35	6/29	68 (37–87)	AL:26; PF:5; SL: 4	IIB: 2; IIC: 3; IIIA: 13; IIIB: 4; pd: 13;	na	na	Schoellner cup; Zimmer Biomet
Barrientos-Ruiz et al. [14]	10	6/4	56 (33–77)	PT, ST	na	nr	S: 7; ST: 2 hematoma: 1	- Stanmore (Worldwide Ltd, Elmstree, UK) - Socincer custom-made (Gijón, Spain)
De Paolis et al. [15]	45	24/21	47 (17–79)	PT	na	P1–P2: 7; P2: 17; P2–P3: 18; P1–P2–P3: 3	CS: 29; OS: 9; ST: 3; AS: 1; AL: 2; ES: 1	nr
Desbonnet et al. [16]	48	24/24	74.1 (53–96)	R	nr	na	na	Integra Lepine (Genay France)
Willemse et al. [17]	24	5	69.5 (39–86)	AL: 20 cases; AF: 1; AL: 2; RD: 1	nr	na	na	McMinn
Eisler et al. [18]	25 (26 hips)	5	59 (33–85)	25 R 1 T	nr	na	na	McMinn

*Pts* patients, *M* male, *F* female, *ST* secondary tumors, *PF* periprosthetic fractures, *AF* acetabular fracture, *AL* aseptic loosening, *SL* septic loosening, *nr* not reported, *na* not applicable, *CS* chondrosarcoma, *OS* osteosarcoma, *ES* ewing sarcoma, *EH* Epithelioid Haemangioendothelioma, *HT* Haematopoietic tumors, *R of T* revision of tumoral cases, *dedif* dedifferentiated, *MVS* Malignant Villonodular Synovitis, *MM* Multiple Myeloma, *PUS* Pleomorphic Undifferentiated Sarcoma, *SNOS* Sarcoma Not Otherwise Specified, *PMT* Phosphaturic Mesenchymal Tumor

### Indications

Stemmed acetabular prosthesis was implanted in 221 cases for primary or secondary tumors or revision of reconstructions after pelvic tumor resections; the second indication was aseptic loosening of previous prosthesis in 79 cases, acetabular fractures in 26 cases, periprosthetic fractures in 17 cases and septic loosening in 12 cases; in 48 cases reported by Desbonnet et al., and in 25 cases reported by Eisler et al., the specific cause of revision was not described (Table 1) [16, 18].

### Histologies

Chondrosarcoma: 93 cases; Metastasis: 66 cases; Osteosarcoma: 26 cases; Ewing Sarcoma: 14 cases; hematopoietic tumors: 7; not specified sarcoma: 8; other: 11. Bus et al., reported 47 patients but only 46 histologies (Table 1).

### Hardware

**Fig. 2:**
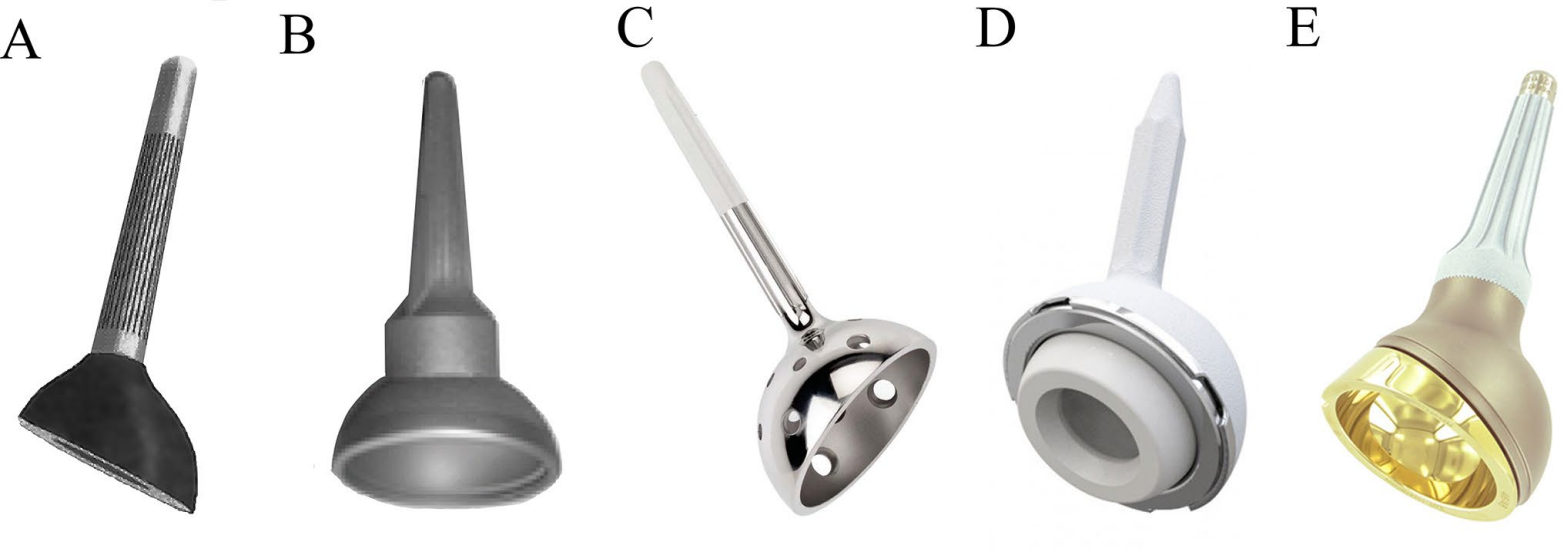
The cone prostheses used in the series included in present review: **A** McMinn cone prosthesis. **B** Schoellner cup cone prosthesis (Zimmer-Biomet). **C** Stanmore cone prosthesis. **D** Integra cone prosthesis (Lepine). **E** Lumic Cone Prosthesis (Implant-Cast)

Several prostheses are present in literature, although the first models are no longer commercially available. Although the principles are similar, they present different shapes and characteristics. In Fig. 2, the main SAC prostheses are showed.

The Integra-Lepine^®^ cone-prosthesis (Genay, France) was used in 48 cases; the coned Hemi-Pelvis Stanmore prosthesis (Stanmore, UK) was used in 54 cases; the McMinn original prosthesis was used in 50 cases; the Lumic-Implant Cast^®^ cone-prosthesis (Hamburg, Germany) in 68 cases; the Schoellner-Zimmer cone-prosthesis (Freiburg, Germany) was implanted in 35 cases; METS Coned-Stryker (Newbury, UK) was used in 22 cases; the coned prosthesis was not identified in 45 cases (Table 1).

### Surgeries

The approach used was the standard Enneking for tumoral resection cases and the posterolateral approach for the other indications.

Cement or grafts were used to fill the gaps and increase the bone stock.

The operative time was obviously different between tumoral cases and revision cases; data for tumor cases were only available from the papers of Erol et al., Barrientos-Ruiz et al., and De Paolis et al., for a total of 76 cases with an average operative time of 347.6 min [10, 14, 15]; the average duration for degenerative and revision cases was reported by McMahon et al. and Stihsen et al. for 56 cases and 139.2 min [9, 13].

Data are not informative for mixed series.

### Complications

218 episodes of complications are reported in the present review; aseptic loosening and implant failure are the most frequent complications, reported in 67 of 428 cases (15.6%), followed by deep infection and dislocation, reported in 48 (11.2%) and 42 (9.8%) cases, respectively. No statistically significative differences were found regarding the different prostheses and specific complications.

Considering the 48 infections, 36 episodes occurred in tumoral cases (16.3%) and 12 in degenerative cases (5.8%). The difference was statistically significative (*P* = 0.0006).

Wound necrosis was reported in 12 cases (2.8%), periprosthetic fracture was reported in 7 cases (1.6%). Lymphedema was only reported in the paper of Fujiwara et al. in 5 out of 54 cases [7]; length discrepancy, reported in 11 out of 54 cases by Fujiwara et al. and in all 10 cases reported by Barrientos-Ruiz et al. [7, 14]; Issa et al., in 2020 reported an average discrepancy of 9.5 mm, Erol et al. reported an average discrepancy of 2 cm [8, 10].

### Follow-up

The weighted average follow-up was 44.8 months.

### Functional results

The average MSTS score expressed on 156 tumoral cases was 66.4%. De Paolis et al. reported 11 excellent, 14 good, 5 fair, and 2 poor results following the MSTS score on 45 tumoral cases (13 not evaluated) [15]. Considering just the papers in which information about walking is reported (Issa et al. and Barrientos-Ruiz et al.), out of 26 patients, 9 patients needed 2 crutches, 8 patients 1 crutch and 9 patients were able to walk without crutches [8, 14].

Data regarding functional results for degenerative cases were quite fragmented, because every group expressed results in a different way.

The 5-years implant survival, reported for 172 patients, was 74.8%; the percentage is 73.5% if we consider just the 137 tumoral cases; actually, Stihsen et al., reported a 5-years implant survival of 78% expressed on non-oncological cases [13].

## Discussion

Reconstructions of acetabular defects remain a challenging problem for orthopedic surgeons; the ideal technique should assure joint function and restore bone stock, moreover in young patients considering possible future revisions.

The use of SACs is not so common; indeed, few papers are available in literature and they are quite limited to reconstruction after wide tumoral resection or after intralesional surgery; nevertheless, their use should also be considered for revisions of degenerative cases, because they allow for primary stability and bone stock recovery considering the possible use with massive bone grafts.

Actually, they could also be indicated as choice in very selected primary cases, as in acetabular fractures in older patients; indeed, in 2017, McMahon and Cusick published a series of six hips in five patients affected by traumatic acetabular fractures, successfully treated with an iliac stem cup prosthesis; they applied the concepts normally used for femoral fractures in the elderly to allow early weight bearing, reducing bedrest [19]. They do not report any complications related to the surgeries but just to the traumas. After that, the same group published their results extended to 22 cases, which were reported in the present review [9].

More than half of the cases in the present review were primary or secondary bone tumors; the most frequent histologies reflect the specific epidemiology of primary tumors in the pelvis: the most common were chondrosarcoma, for which wide resection is considered the gold standard treatment and the only possibility to cure the patient [20] followed by Ewing sarcoma and Osteosarcoma [20–22].

Reconstruction after resection or intralesional curettage of secondary tumors is also common in the present review but it becomes rare if we take the high diffusion of acetabular metastases into account; nevertheless, few of them present surgical indication.

**Fig. 3:**
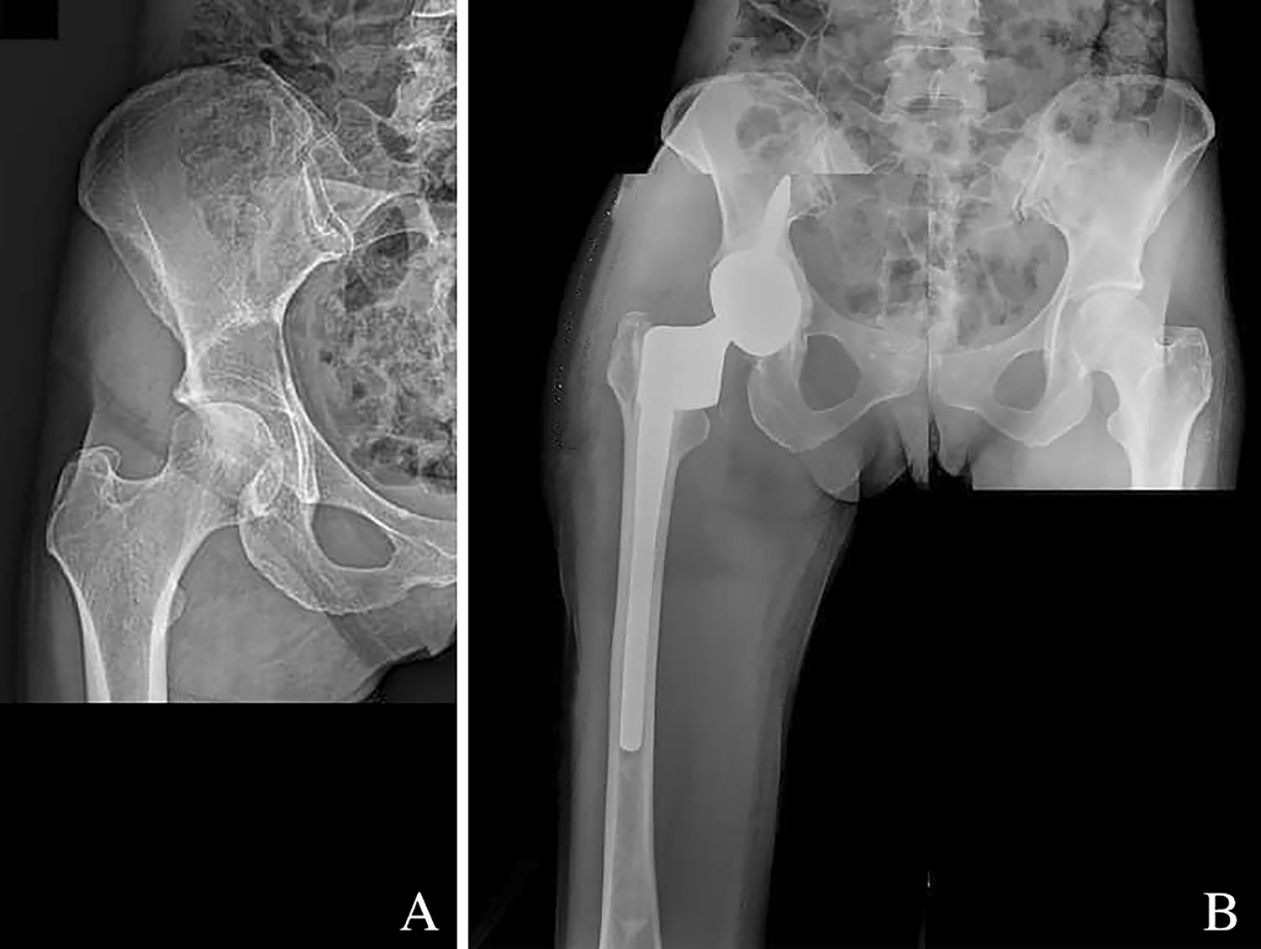
**A** Preoperative X-ray showing a consistent osteolysis caused by a metastasis from breast cancer in a 55-year-old woman; **B** post operative X-ray showing the use of a SAC acetabular prosthesis used to reconstruct the hip after curettage of the cavity

In case of a solitary metastasis from a non-aggressive histology, wide resection can also be considered suitable, otherwise, curettage and local adjuvant can be helpful to decrease local tumor aggressiveness, restore joint stability and allow weightbearing (Fig. 3).

SAC prostheses can also be considered a valid alternative in case of loosening of previously implanted hip prosthesis (79 cases), in case of acetabular fractures to allow precocious weight baring (26 cases), in case of periprosthetic fractures (17 cases) or septic loosenings.

Actually, SAC is characterized by a consistent primary stability, mainly based on the iliac stem and therefore not influenced by the quality and quantity of the surrounding bone; indeed, aseptic loosening and implant failure are the main complications that occurred in 15.6% of cases.

The surgical technique should not be considered difficult for an expert hip surgeon.

In case of reconstruction after tumor removal, the surgical approach usually guarantees a wide exposure and an easy insertion of the SAC; the Enneking access is the most frequently used yet this approach can cause wound flap devascularization with the related complication and lymphoedema, as reported by Fujiwara et al. in 5 out of 54 pelvis resections. In case of revision in degenerative cases, the posterolateral approach is suggested, because it allows an easier identification of the ischiatic notch and a correct positioning of the stem; currently, navigation tools are a valid aid to assure optimal cup orientation [7].

Issa et al., in a recent series of 16 degenerative cases, used the posterolateral approach in 4 cases and the transtrochanteric approach in 12 cases; this should guarantee an easier visualization of the acetabulum; nevertheless, the osteotomy of the greater trochanter could add further complications [8].

Correct stem positioning is quite important to obtain primary stability; in case a massive acetabular graft is used, it is important that a sufficient part of the cup stem is inserted into the patient’s bone. Fujiwara et al., recently demonstrated that it is extremely important to insert the cone into the bone at least for half of its length; in these cases, the aseptic loosening rate is lower than when the cone is more “naked” [7].

No specific indication is present about the necessity or not to cement the acetabular prosthesis; if the stem is inserted with a press-fit technique, the bone gap should be filled with bone graft or cement, based on the cup’s characteristics, the surgical indication, and if reconstruction is done after a degenerative case, after tumor resection or after intralesional tumoral surgery.

Indeed, after degeneration, restoring the bone stock is important so bone grafting is more indicated; after resection of a primary tumor, reconstruction with a massive graft (composite prosthesis) is suggested; after intralesional surgery for a secondary tumor, the use of cement is recommended to increase primary stability and decrease the risk of cup mobilization in case of osteolysis progression.

Complications are quite frequent; they are related not only to the entity of the surgery but also to the patients, who often have several comorbidities.

The most frequent complication is aseptic loosening and implant failure (16.2%). This rate could be deemed more than acceptable, moreover considering that SAC prosthesis is usually reserved for patients who already underwent more than one revision: Kuijpers et al. reported a 5-years failure rate of revised hip prostheses of 22% in 1037 patients younger than 55 [23]; Lie et al. [24], reported a 10-years failure rate of 26% on 4762 hip revisions.

Deep infection and dislocation are the second and third most common complications; they are more often correlated to tumoral cases in which the extended approach needed for tumoral removal, the related devascularization of the flap and the damage of contention systems can be responsible for these adverse events.

Furthermore, this difference can widely be justified by the different duration of the surgeries, although few papers reported the length of surgeries.

Actually, SAC prosthesis can be considered a strategy to decrease the infection risk in pelvic tumor reconstruction, because it is less complex than other reconstruction techniques. Indeed, Angelini et al. reported an infection rate of 23.6% in 129 cases of pelvic tumors reconstructed with several techniques [25].

The dual mobility cup is acknowledged to decrease the dislocation risk and its use is becoming routinary in oncological patients after proximal femur resection [26]. Indeed, Bus et al., in 2016, published a series of 47 patients who underwent acetabular tumor resection and reconstruction with a stem prosthesis, evidencing how the risk of dislocation was consistently lower for patients with a dual-mobility cup than for patient without it (4% against 39%) [12].

The use of a reattachment tube could also be considered a good strategy to decrease the risk of dislocation, moreover in case of consistent resection and bone loss; however, a possible increase of infection risk has to be taken into account [27].

Other possible complications are leg length discrepancy, related to the intrinsic difficulty in understanding the correct leg length when bone loss is consistent; Issa et al., in 2020, reported a mean discrepancy of 9.5 mm between the two legs after surgeries, whereas Erol et al. reported an average discrepancy of 2 cm, clinically well tolerated [8, 10].

Another possible complication is intraoperative fracture during SAC prosthesis insertion; in the present review, it was only reported in seven cases, but this is probably underestimated; the complication could be minimized by reaming the ischiatic isthmus; moreover, several techniques were described to reduce the risk of mispositioning, as using a k-wire as a reaming guide and CT-guided navigation [7, 12, 16].

Fujiwara et al. reported five cases of lymphedema out of 54 patients, but this complication is often associated to the Enneking and the ileo-inguinal approaches [7]; the other authors do not mention it but maybe because they did not consider it.

The average MSTS score based on data from 156 tumoral cases was 66.4%; corresponding to a good result; actually, results reported from De Paolis et al. are quite concordant with our analysis [15]. The patients able to walk without crutches, with one crutch or with two crutches are quite equally distributed.

Functional results for degenerative cases are not reported, because data are quite fragmented since every author group expressed results in a different way; nevertheless, it is likely to think that functional recovery should be better in these patients, although the expectations are higher.

The 5-years implant survival (74.8% of 172 patients) was quite high both for oncological cases who undergo very extended resections and for degenerative cases, also because SAC is often used after more than one revision.

Nevertheless, the present paper presents several limitations;

- Selected articles have been published over a long period of time so today's results may be better and associated with improved materials but the average results could underestimate the current results; moreover, the included prostheses present different characteristics and shape; this introduces a confounding factor in present evaluation.
- Functional evaluation: the MSTS score is appointed by the surgeon, who could assign a better value for personal belief.
- Complications not reported in the papers were considered not occurred but, unfortunately, this could not always be true.
- The follow-up of single studies is not lengthy enough to understand long-term results; no information is available about complications onset afterwards.

Data regarding SAC prosthesis are quite rare in literature; no prospective studies with comparisons to other reconstruction techniques are available so their use is mainly based on the experience of single centers. While data are more consistent and supported for tumors, it is quite fragmented for studies using the same methods to revise hip prostheses implanted for degenerative problems. Nevertheless, preliminary results are encouraging and SAC prostheses can be considered a valid alternative both for tumoral and degenerative revision cases. Prospective randomized studies are advocated to value results confronted with other alternative techniques.
